# Sequence comparison of the mitochondrial genomes of five brackish water species of the family Neritidae: Phylogenetic implications and divergence time estimation

**DOI:** 10.1002/ece3.8984

**Published:** 2022-06-06

**Authors:** Jing Miao, Jiantong Feng, Xiaojuan Liu, Chengrui Yan, Yingying Ye, Jiji Li, Kaida Xu, Baoying Guo, Zhenming Lü

**Affiliations:** ^1^ 71233 National Engineering Research Center for Facilitated Marine Aquaculture Zhejiang Ocean University Zhoushan China; ^2^ 12386 Guangdong Provincial Key Laboratory of Marine Biotechnology Shantou University Shantou Guangdong China; ^3^ 71233 Marine Fishery Institute of Zhejiang Province Key Laboratory of Sustainable Utilization of Technology Research for Fishery Resource of Zhejiang Province Zhejiang Ocean University Zhoushan China

**Keywords:** divergence time, mitogenome, Neritid, phylogenetic

## Abstract

Neritids are ancient gastropod species which can live in marine, brackish water, and freshwater environments. In this study, we sequenced and annotated the mitochondrial genomes of five brackish water neritids (i.e., *Clithon corona*, *Clithon lentiginosum*, *Clithon squarrosum*, *Neritina iris*, and *Septaria lineata*). The mitogenomes ranged from 15,618 to 15,975 bp, and all contain 13 protein‐coding genes (PCGs), 22 tRNA genes, and two rRNA genes, with a closed ring structure. We calculated the Ka/Ks values of all 13 PCGs of Neritidae species, all ratios are less than 1, under purification selection. Phylogenetic analysis of the 13 PCGs showed that Neritimorpha is a sister group with Vetigastropoda and Caenogastopoda, genus *Clithon* is a sister group with *Neritina* and *Septaria*. Estimation of divergence time for all species of Neritidae showed that the main differentiation of Neritidae occurred in Cenozoic period (65 Mya), *C*. *corona* and *C*. *lentiginosum* were differentiated in the Cenozoic Neogene, the other three species diverged in the Cenozoic Paleogene. These results will help to better understand the evolutionary position of Neritidae and provide reference for further phylogenetic research on Neritidae species.

## Graphical abstract

Enrich the mitochondrial genome data of Neritidae species. Established the phylogenetic tree of gastropoda. Speculated the separation time of Neritidae species.

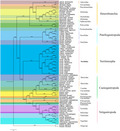

## 1 INTRODUCTION

Neritidae (Gastropoda: Neritimorpha: Cycloneritida) is one of the most diverse taxa in the Neritimorpha (Rafinesque, 1815). At present, there are 16 genera, comprising around 280 species (Hamish et al., 2007), with about 40 species having been found on the southeast coast of China before 2008 (Zhang, 2008). The fossil record of neritids dates back to the late Cretaceous (Kano, 2002), showing ecological radiation and extreme diversity in form. Neritids occur mainly in intertidal zone (Sasaki et al., 2002). They are euryhaline, and can live in marine, brackish water, and freshwater ecosystems, *Nerita* species are almost exclusively found in marine environments, *Clithon* and *Neritina* animals are mostly found in freshwater and brackish water environments (Tan & Clements, 2008). There have been at least five or six evolutionary transitions from hypersaline environments to freshwater in the evolutionary history of Neritidae (Frey, 2010; Holthuis, 1995). However, most freshwater lineages retain a dispersed planktonic marine larval stage, in which adults develop, reproduce in rivers, hatch larvae enter the sea, grow into adults, and return to freshwater in a cycle (Abdou et al., 2015).

The metazoan mitochondrial genome (mitogenome) is a double‐stranded molecular structure in the form of a closed ring. It usually has 37 coding genes, including 13 protein‐coding genes (PCGs), two ribosomal RNA genes (rRNA), 22 transfer ribonucleic acid (tRNA) genes, and a noncoding control region (CR) (Fernández‐Silva et al., 2003; Wolstenholme & David, 1992). The mitogenome is characterized by high conservation, lack of extensive recombination, maternal inheritance, and a high mutation rate (Curole & Kocher, 1999; William et al., 2004). Compared with some gene fragments, such as COI (cytochrome c oxidase subunit 1) and 16S rRNA, mitogenome sequences can better elucidate evolutionary relationships between species, it has been widely used in phylogenetic researches (Zardoya & Meyer, 1996).

Next‐generation sequencing (NGS) have been widely used in phylogenetic analysis, and the study of Neritidae classification has been ongoing for a long period. However, there are insufficient studies on the mitochondrial data and divergence time of neritids. In this study, we chose five neritid species: *Clithon corona*, *Clithon lentiginosum*, *Clithon squarrosum*, *Neritina iris*, and *Septaria lineata*, which can live in both fresh and brackish water environments. After sequencing, assembly, annotation, and analysis of the complete mitogenome, we analyzed their basic characteristics in the five species, calculated the average nonsynonymous to synonymous substitution ratio (Ka/Ks) of 19 Neritidae species, constructed the phylogenetic tree of mitogenomes of Gastropoda to analyze the phylogenetic position and relationship in Neritidae, and speculated the differentiation time of neritids.

## 2 MATREIALS AND METHODS

### 2.1 Sample collection and DNA extraction

Five species of Neritidae *C*. *corona*, *N*. *iris*, *S*. *lineata*, *C*. *squarrosum*, and *C*. *lentiginosum* were collected from the coastal area of Huizhou, Guangdong Province, China (Table 1). The preliminary morphological identification of these samples was carried out by consulting the taxonomic experts of the Marine Biological Museum of Zhejiang Ocean University. Store samples in absolute ethanol, take a small piece of fresh foot tissue to extract total DNA by salting‐out method (Aljanabi & Martinez, 1997), and store at −20°C.

**TABLE 1:** Sampling locations and dates for the five samples

Species name	Sampling date	Sampling location
*Clithon corona*	September 2020	114°55′17.66″E, 22°73′54.44″N
*Clithon lentiginosum*	October 2020	114°72 ′56.06″E, 22°79′94.94″N
*Clithon squarrosum*	October 2020	114°72 ′81.75″E, 22°79′92.06″N
*Neritina iris*	September 2020	114°54′55.86″E, 22°73′86.77″N
*Septaria lineata*	September 2020	114°55 ′61.32″E, 22°73′43.02″N

### 2.2 Mitogenome sequencing, assembly, and annotation

The complete mitogenomes of five species were sequenced on the Illumina Hiseq X Ten platform by Origingene Bio‐pharm Technology Co., Ltd. (Shanghai, China). The Covaris M220 physical method (ultrasonic) was used to fragment the DNA, and the length of the fragments was 300−500 bp. Then, the DNA fragments were purified to construct a sequencing library. The Illumina HiSeq™ platform was used for sequencing after library quality inspection, and a 10 Gb data volume was used for sequencing. Data quality control was performed by Trimmomatic v0.39 (http://www.usadellab.org/cms/index.php?page=trimmomatic) (Bolger et al., 2014), filter out low‐quality reads, duplicated reads, sequences with an “N” rate greater than 10%, and sequencing linker sequences. Clean data with high quality was obtained and the reads of the five species were de novo assembled using NOVOPlasty assembly software (https://github.com/ndierckx/NOVOPlasty) (Dierckxsens et al., 2017). The stack cluster was compared with reference genome in the GenBank database, and majority of the mitogenome sequence information was obtained. Then, the online software MITOS (http://mitos.bioinf.uni‐leipzig.de/index.py) was used for structural and functional annotation and manual correction (Bernt et al., 2013), the complete mitogenome was finally obtained. Sequenced mitogenomes were uploaded to GenBank database at the National Center for Biotechnology Information (NCBI).

### 2.3 Sequence analysis

Circular genome visualization of five species was generated using the online CGView server (http://cgview.ca/) (Stothard & Wishart, 2005). The nucleotide composition and relative synonymous codon usage (RSCU) of each protein‐coding gene were calculated using MEGA‐X (Kumar et al., 2018). The AT‐skew and GC‐skew computation formulae were as follows: AT‐skew = (A − T)/(A + T), GC‐skew = (G − C)/(G + C) (Hassanin et al., 2005). The Ka/Ks ratio of the five mitogenomes was estimated using DnaSP 6.0 (Rozas et al., 2017).

### 2.4 Phylogenetic analyses

The phylogenetic analyses of the five species were performed using the sequences of complete mitogenomes from 81 species (Table 2). A total of 74 Gastropoda species from Neritimorpha, Vetigastropoda, Caenogastropoda, Patellogastropoda, and Heterobranchia were downloaded from GenBank (https://www.ncbi.nlm.nih.gov/genbank/) for phylogenetic analysis. Two Veneridae species *Dosinia troscheli* (NC_037917) and *Dosinia japonica* (NC_038063) were used as outgroups. The sequence of the 13 PCGs of each specie were identified using DAMBE 7 (Xia, 2018), the PCGs of each sample were concatenated together in the same order, the tree building sequence set was obtained by combining them in a unified sequence. The PCGs sequences of these 81 species were aligned using ClustalW of MEGA‐X. Nucleotide substitution saturation was analyzed using DAMBE 7 to evaluate whether these sequences were suitable for phylogenetic tree construction.

**TABLE 2:** List of Gastropoda species used in phylogenetic analysis with their GenBank accession numbers, and five newly sequenced Neritid species were marked with^✽^

Subclass	Family	Species	Size (bp)	Accession no.
Heterobranchia	Placobranchidae	*Elysia cornigera*	14,118	NC_035489
		*Plakobranchus ocellatus*	14,173	AP014544
	Aplysiidae	*Aplysia dactylomela*	14,128	DQ991927
		*Aplysia kurodai*	14,131	KF148053
	Onchidiidae	*Peronia peronii*	13,968	JN619346
		*Platevindex mortoni*	13,991	NC_013934
	Ellobiidae	*Myosotella myosotis*	14,246	NC_012434
		*Auriculinella bidentata*	14,135	JN606066
		*Ellobium chinense*	13,979	NC_034292
		*Carychium tridentatum*	13,908	KT696545
		*Ovatella vulcani*	14,274	JN615139
	Volvatellidae	*Ascobulla fragilis*	14,745	AY345022
	Siphonariidae	*Siphonaria gigas*	14,514	NC_016188
		*Siphonaria pectinata*	14,065	NC_012383
	Polyceridae	*Nembrotha kubaryana*	14,395	NC_034920
		*Roboastra europaea*	14,472	NC_004321
		*Notodoris gardineri*	14,424	NC_015111
Patellogastropoda	Nacellidae	*Nacella clypeater*	16,742	KT990124
		*Nacella magellanica*	16,663	KT990125
		*Nacella concinna*	16,761	KT990126
		*Cellana grata*	16,181	MW722939
		*Cellana nigrolineata*	16,153	LC600801
		*Cellana radiata*	16,194	MH916651
	Patellidae	*Patella ferruginea*	14,400	MH916654
		*Patella pellucida*	14,949	OU795045.1
		*Patella vulgata*	14,808	MH916653
	Pectinodontidae	*Bathyacmaea lactea*	18,446	MW309841
		*Bathyacmaea nipponica*	16,792	MF095859
Caenogastropoda	Muricidae	*Ceratostoma burnetti*	15,334	NC_046569
		*Ceratostoma rorifluum*	15,338	MK411750
		*Ocinebrellus falcatus*	15,326	NC_046052
		*Boreotrophon candelabrum*	15,265	NC_046505
	Conidae	*Conus betulinus*	16,240	NC_039922
		*Conus tulipa*	15,756	KR006970
	Naticidae	*Euspira gilva*	15,315	NC_046593
		*Euspira pila*	15,244	NC_046703
		*Mammilla kurodai*	15,309	NC_046596
	Pomatiopsidae	*Oncomelania quadrasi*	15,184	LC276227
	Muricidae	*Chicoreus torrefactus*	15,359	NC_039164
		*Indothais lacera*	15,272	NC_037221
		*Rapana venosa*	15,272	NC_011193
		*Menathais tuberosa*	15,294	NC_031405
	Clavatulidae	*Turricula nelliae spuria*	16,453	MK251986
		*Turritella bacillum*	15,868	NC_029717
Vetigastropoda	Turbinidae	*Angaria neglecta*	19,470	NC_028707
		*Astralium haematragum*	16,310	NC_031858
		*Bolma rugosa*	17,432	NC_029366
		*Lunella granulate*	17,190	NC_031857
	Tegulidae	*Chlorostoma argyrostomum*	17,780	KX298892
		*Omphalius nigerrimus*	17,755	NC_031862
		*Tegula brunnea*	17,690	NC_016954
		*Tegula lividomaculata*	17,375	NC_029367
	Haliotidae	*Haliotis iris*	17,131	NC_031361
	Trochidae	*Gibbula umbilicalis*	16,277	NC_035682
		*Monodonta labio*	16,440	MK240320
		*Stomatella planulata*	17,151	NC_031861
		*Umbonium thomasi*	15,998	MH729882
	Peltospiridae	*Chrysomallon squamiferum*	15,388	AP013032
		*Gigantopelta aegis*	15,176	MT312227
	Phasianellidae	*Phasianella solida*	16,698	NC_028709
Neritimorpha	Neritidae	Clithon corona*	15,975	MZ189741
		Clithon lentiginosum*	15,885	MZ152905
		Clithon squarrosum*	15,905	MZ297477
		*Clithon oualaniense*	15,705	MT568501
		*Clithon retropictus*	15,802	NC_037238
		*Clithon sowerbianum*	15,919	MT230542
		Neritina iris*	15,618	MZ189742
		*Neritina violacea*	15,710	KY021066
		Septaria lineata*	15,697	MZ315041
		*Nerita albicilla*	15,314	MK516738
		*Nerita balteata*	15,571	MN477253
		*Nerita chamaeleon*	15,716	MT161611
		*Nerita undata*	15,583	MN477254
		*Nerita versicolor*	15,866	KF728890
		*Nerita fulgurans*	15,343	KF728888
		*Nerita tessellata*	15,741	KF728889
		*Nerita japonica*	15,875	MN747116
		*Nerita melanotragus*	15,261	GU810158
		*Nerita yoldii*	15,719	MK395169

The Bayesian inference (BI) method of the program MrBayes 3.2.7a (Ronquist et al., 2012) and the maximum likelihood (ML) method of IQ‐tree 2.1.3 (Minh et al., 2020) were used to analyze the phylogenetic relationships. The Bayesian method model measurement firstly used PAUP 4 (Swofford, 2002) software for format conversion, and then used MRMTGUI (Nuin, 2005) software to associate PAUP 4, ModelTest 3.7 (Posada, 2005) and MRModelTest 2.3 (Nylander et al., 2004) programs to determine the best alternative model under the Akaike information criterion (AIC) as GTR + I + G. BI analysis was performed using two Markov chain Monte Carlo (MCMC) run with 2 million generations, and sampling was performed once every 1000 generations. the first 25% of trees were discarded as burn‐in, and convergence for independent operation was evaluated using the mean standard deviation of the splitting frequency (<0.01).

The ML tree best fit replacement model (GTR + F + I + G4) selected by Bayesian information criterion (BIC) using ModelFinder (Kalyaanamoorthy et al., 2017), setting the boot copy number with 1000 ultra‐fast bootstraps in order to reconstruct the consensus tree. Finally, the phylogenetic tree was viewed, edited, and visualized using the Figtree 1.4.4 (Rambaut, 2018) software.

### 2.5 Estimation of divergence times

Twenty Neritimorpha species were chosen to estimate the divergence time, including 19 species in Neritidae, and *Pleuropoma jana* from family Helicinidae. Based on the 13 PCGs datasets at the nucleotide level, we used a Bayesian tree as the framework to estimate the divergence time of the Neritimorpha species. The analysis was performed using the BEAST 1.8.4 software (Drummond et al., 2012). We used an uncorrelated lognormal relaxed molecular clock model. The Yule process was used for the tree prior, and divergence time calibration was used for the distribution of standard points of fossils. The MCMC was analyzed twice, with 100 million generations each, and sampled once every 1000 generations. Ten percent of samples discarded as a burn‐in by TreeAnnotator 1.8.4 package in BEAST. Tracer 1.6 (Rambaut & Suchard, 2014) was used to verify chain convergence and majority values exceed the effective sample size (ESS) of 200. Calibration points of divergent time was determined from the reported fossil record. At present study, we specify two calibration points as priors and use a normal distribution. The first calibration point, based on the Mesozoic Triassic period, is that *Pleuropoma jana* is limited from 235 to 223 million years ago (Mya) (Uribe, Kano, et al., 2016). Based on the Mesozoic Cretaceous *Nerita melanotragus* fossil record (95–80 Mya) (Postaire et al., 2014), 80 Mya was set as another calibration point with a standard deviation of 2.0. A public repository of time scale information on evolution Timetree (http://www.timetree.org/) (Hedges et al., 2006), we used reported results to verify the accuracy of the divergence time (Frey & Vermeij, 2008; Postaire et al., 2014; Uribe et al., 2019). Finally, the Figtree 1.4.4 (Rambaut, 2018) software was used to edit the divergence time tree.

## 3 RESULTS AND DISCUSSION

### 3.1 Genome structure, composition, and skewness

**FIGURE 1:**
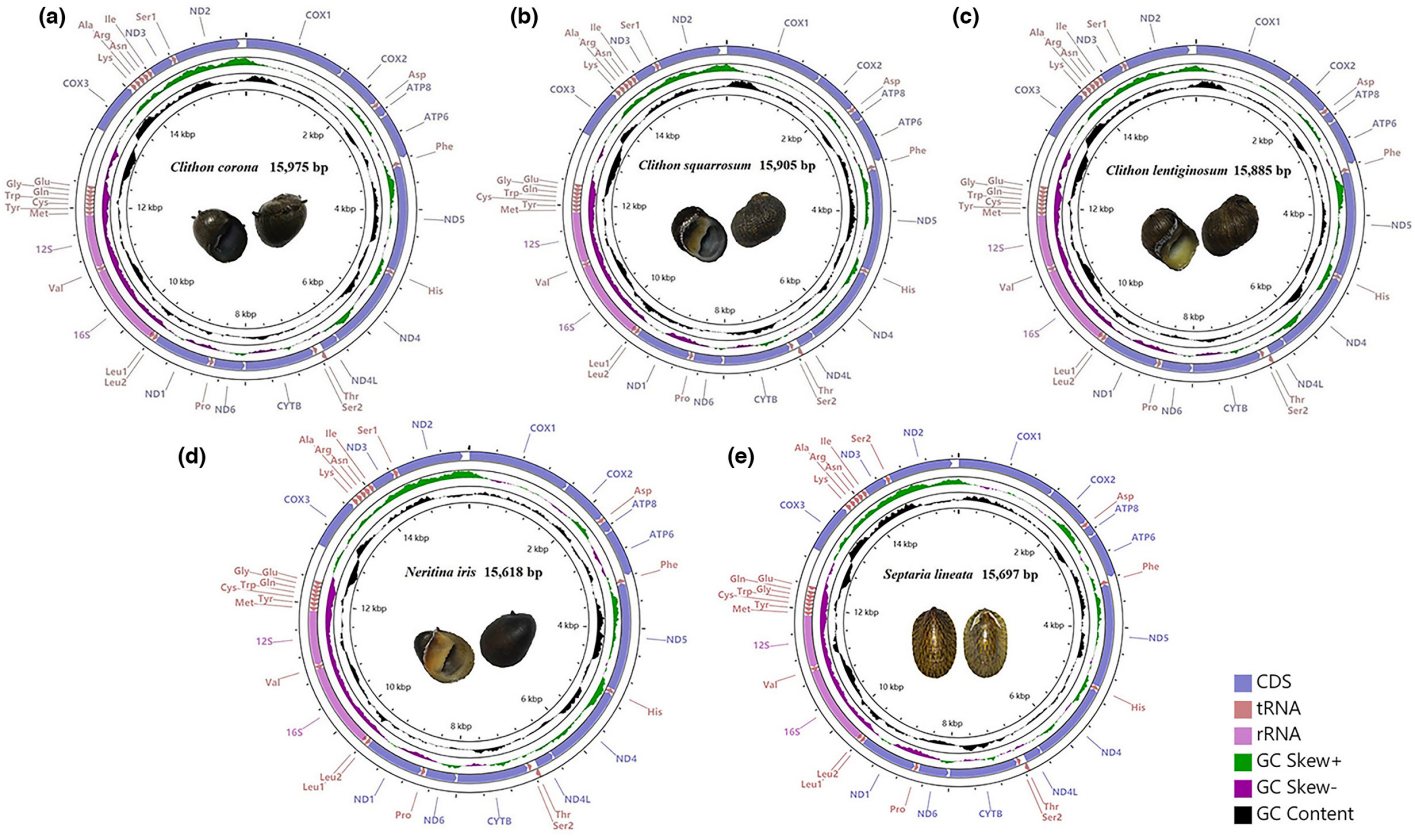
Complete mitogenome map of five neritid species

The complete mitogenome sequences of the five Nerita species consist of 15,975 bp (*C*. *corona*), 15,885 bp (*C*. *lentiginosum*), 15,905 bp (*C*. *squarrosum*), 15,618 bp (*N*. *iris*), and 15,697 bp (*S*. *lineata*), the smallest being for *N*. *iris* and the largest for *C*. *corona*. The GenBank accession numbers are MZ189741, MZ152905, MZ297477, MZ189742, and MZ315041, respectively (Figure 1). They are all closed, circular, double‐stranded DNA molecules, containing 37 typical coding genes, including 13 PCGs, 22 tRNA genes, two rRNA genes (12S rRNA and 16S rRNA), and a control region (CR). Among them, 15 genes (seven PCGs and eight tRNA genes) are located on the heavy chain, while the others were located on the light chain (Figure 1). The longest gene was ND5, with a length of 1702 to 1717 bp, and the shortest was the ATP8 gene, with a consistent length of only 165 bp (Table 3).

**TABLE 3:** Composition and skewness in the mitogenomes of five neritid species

Cc/Cl	Size(bp)	A(%)	T(%)	G(%)	C(%)	A+T(%)	AT‐skew	Size(bp)	A(%)	T(%)	G(%)	C(%)	A+T(%)	AT‐skew
Mitogenome	15,975	30.66	34.06	20.24	15.04	64.72	−0.05	15,885	30.61	34.16	20.15	15.08	64.77	−0.05
COI	1548	22.29	39.86	22.22	15.63	62.15	−0.28	1548	22.22	40.25	22.16	15.37	62.47	−0.29
COII	690	28.12	36.52	20.58	14.78	64.64	−0.13	690	27.68	35.80	20.87	15.65	63.48	−0.13
ATP8	165	29.70	43.64	18.18	8.48	73.34	−0.19	165	28.48	44.24	19.39	7.88	72.72	−0.22
ATP6	699	22.17	42.49	19.89	15.45	64.66	−0.31	702	22.22	42.31	19.80	15.67	64.53	−0.31
COIII	780	20.64	39.23	23.85	16.28	59.87	−0.31	780	20.26	38.97	24.10	16.67	59.23	−0.32
ND3	354	20.62	44.35	24.01	11.02	64.97	−0.37	354	20.62	44.35	24.01	11.02	64.97	−0.37
ND1	933	26.80	36.01	16.51	20.69	62.81	−0.15	933	26.69	36.87	17.04	19.40	63.56	−0.16
ND5	1717	28.77	33.95	14.50	22.77	62.72	−0.08	1702	29.38	34.96	13.22	22.44	64.34	−0.09
ND4	1233	27.66	36.09	14.44	21.82	63.75	−0.13	1254	28.07	35.33	14.51	22.09	63.40	−0.11
ND4L	294	28.57	34.69	17.69	19.05	63.26	−0.10	294	29.25	35.37	16.67	18.71	64.62	−0.09
ND6	501	26.55	39.52	13.97	19.96	66.07	−0.20	501	26.55	40.72	14.17	18.56	67.27	−0.21
Cytb	1137	25.42	37.73	16.18	20.67	63.15	−0.19	1137	26.03	37.38	15.92	20.67	63.41	−0.18
ND2	1003	21.44	41.58	24.73	12.26	63.02	−0.32	1003	22.93	40.88	23.23	12.96	63.81	−0.28
tRNAs	1417	31.26	32.53	21.10	15.10	63.79	−0.02	1484	30.80	32.28	21.50	15.43	63.08	−0.02
rRNAs	2193	36.98	30.60	16.87	15.55	67.58	0.09	2193	36.89	30.60	16.78	15.73	67.49	0.09
PCGs	11,054	25.18	38.09	18.64	18.08	63.27	−0.20	11,063	25.45	38.20	18.36	18.00	63.65	−0.20

Ni/Cs	Size(bp)	A(%)	T(%)	G(%)	C(%)	A+T(%)	AT‐skew	Size(bp)	A(%)	T(%)	G(%)	C(%)	A+T(%)	AT‐skew
Mitogenome	15,618	29.89	34.45	20.85	14.80	64.34	−0.07	15,905	30.86	34.06	20.24	14.84	64.92	−0.05
COI	1548	22.16	40.89	21.77	15.18	63.05	−0.30	1548	22.09	39.99	22.55	15.37	62.08	−0.29
COII	690	25.94	35.94	22.32	15.80	61.88	−0.16	690	27.83	36.81	20.72	14.64	64.64	−0.14
ATP8	165	26.67	40.61	22.42	10.30	67.28	−0.21	165	31.52	40.61	17.58	10.30	72.13	−0.13
ATP6	702	22.08	41.60	19.37	16.95	63.68	−0.31	702	21.79	41.74	20.80	15.67	63.53	−0.31
COIII	780	20.77	40.38	23.85	15.00	61.15	−0.32	780	21.28	40.64	23.08	15.00	61.92	−0.31
ND3	354	20.90	44.92	24.01	10.17	65.82	−0.36	354	21.75	43.50	23.16	11.58	65.25	−0.33
ND1	933	27.97	35.69	16.40	19.94	63.66	−0.12	933	26.47	35.91	17.47	20.15	62.38	−0.15
ND5	1716	29.78	32.63	13.52	24.07	62.41	−0.05	1717	29.18	33.49	14.09	23.24	62.67	−0.07
ND4	1323	28.72	34.62	13.53	23.13	63.34	−0.09	1254	28.31	36.60	13.88	21.21	64.91	−0.13
ND4L	294	32.65	31.29	15.31	20.75	63.94	0.02	294	28.23	35.37	17.35	19.05	63.60	−0.11
ND6	495	28.69	36.36	13.74	21.21	65.05	−0.12	501	26.15	39.52	14.77	19.56	65.67	−0.20
Cytb	1137	27.44	35.80	15.22	21.55	63.24	−0.13	1137	25.68	36.85	15.74	21.72	62.53	−0.18
ND2	1003	22.43	41.58	24.53	11.47	64.01	−0.30	1003	22.83	41.67	23.83	11.67	64.50	−0.29
tRNAs	1471	30.80	31.61	22.16	15.43	62.41	−0.01	1417	31.12	32.67	21.38	14.82	63.79	−0.02
rRNAs	2204	35.53	30.54	17.60	16.33	66.07	0.08	2197	36.55	30.45	17.25	15.75	67.00	0.09
PCGs	11,140	25.89	37.35	18.23	18.53	63.24	−0.18	11,078	25.46	38.02	18.51	18.01	63.48	−0.20

Sl	Size(bp)	A(%)	T(%)	G(%)	C(%)	A+T(%)	AT‐skew
Mitogenome	15,697	31.39	34.36	19.32	14.92	65.75	−0.05
COI	1548	23.71	40.18	20.41	15.70	63.89	−0.26
COII	690	27.25	37.10	21.30	14.35	64.35	−0.15
ATP8	165	30.30	43.64	16.97	9.09	73.94	−0.18
ATP6	702	23.79	42.02	19.66	14.53	65.81	−0.28
COIII	780	21.41	40.13	23.46	15.00	61.54	−0.30
ND3	354	21.75	43.22	24.01	11.02	64.97	−0.33
ND1	933	27.55	37.08	16.51	18.86	64.63	−0.15
ND5	1716	29.08	35.20	14.22	21.50	64.28	−0.10
ND4	1323	28.57	37.87	13.98	19.58	66.44	−0.14
ND4L	294	31.63	35.37	15.65	17.35	67.00	−0.06
ND6	495	31.31	39.80	13.33	15.56	71.11	−0.12
Cytb	1137	25.77	37.82	15.92	20.49	63.59	−0.19
ND2	1003	25.12	41.18	21.93	11.76	66.30	−0.24
tRNAs	1478	31.12	31.94	21.65	15.29	63.06	−0.01
rRNAs	2204	36.07	31.58	16.92	15.43	67.65	0.07
PCGs	11,140	26.42	38.65	17.89	17.04	65.07	−0.19

In the five mitogenomes at present study, the average AT content was higher than CG, with a bias of 64.90%. The average AT‐skew was −0.0545, and GC‐skew was 0.1486 (Table 4). The base content of As was lower than that of Ts, and the base content of Gs was higher than that of Cs. In general, the average content of each species in the complete mitogenome was T > A > G > C (Table 3), which is consistent with the reported complete neritids mitogenomes (Arquez et al., 2014; Feng et al., 2020, 2021).

**TABLE 4:** Size and skewness in the mitogenomes of five neritid species

Species	Size(bp)	mitogenome			Size(bp)	PCGs		
		A + T%	AT‐skew	GC‐skew		A + T%	AT‐skew	GC‐skew
*Clithon corona*	15,975	64.72	−0.0525	0.1473	11,054	63.27	−0.2042	0.0153
*Clithon lentiginosum*	15,885	64.77	−0.0548	0.1438	11,063	63.65	−0.2004	0.0099
*Clithon squarrosum*	15,905	64.92	−0.0492	0.1540	11,078	63.48	−0.1980	0.0138
*Neritina iris*	15,618	64.34	−0.0708	0.1697	11,140	63.24	−0.1813	−0.0081
*Septaria lineata*	15,697	65.75	−0.0452	0.1284	11,140	65.07	−0.1880	0.0244
Average	15,816	64.90	−0.0545	0.1486	11,095	63.74	−0.1944	0.0111

Species	Size(bp)	tRNA			Size(bp)	rRNA		
		A + T%	AT‐skew	GC‐skew		A + T%	AT‐skew	GC‐skew
*Clithon corona*	1417	63.79	−0.0199	0.1657	2193	67.58	0.0945	0.0408
*Clithon lentiginosum*	1484	63.08	−0.0235	0.1642	2193	67.49	0.0932	0.0323
*Clithon squarrosum*	1417	63.79	−0.0243	0.1813	2197	67.00	0.0910	0.0455
*Neritina iris*	1471	62.41	−0.0131	0.1790	2204	66.07	0.0755	0.0374
*Septaria lineata*	1478	63.06	−0.0129	0.1722	2204	67.65	0.0664	0.0463
Average	1467	63.23	−0.0187	0.1725	2198	67.16	0.0841	0.0405

### 3.2 Protein‐coding genes and codon usage

The mitogenome of the Neritidae in this study contains 13 PCGs, including a cytochrome b (Cyt *b*), two ATPases (ATP6 and ATP8), three cytochrome oxidases (COI–III), and seven NADH dehydrogenases (ND1–6 and ND4L). The length of the PCGs in these five species is between 11,054 and 11,140 bp (Table 3). The base composition of these species also showed a high AT bias, with the highest AT content being seen in *S*. *lineata*, at 65.75%. The AT bias values of each species were negative, in addition to *N*. *iris* at −0.07, the values of the other four species are −0.05, with the T base content being higher than that of the A base. In these five neritid species, the start codon was ATN, almost all genes initiated with ATG, and only a few genes initiated with ATA (Table 5). The majority of the 13 PCGs terminated with TAG or TAA as stop codons, and some of the PCGs terminated with T as an incomplete codon, which was often found in ND2 and ND5. This incomplete stop codon was usually supplemented during transcription to obtain a complete stop codon T(AA) (Ojala et al., 1981).

**TABLE 5:** Start and stop codons for PCGs of five neritid species

gene	Start codon/stop codon				
	Cc	Cs	Cl	Ni	Sl
COI	ATG/TAG	ATG/TAA	ATG/TAG	ATG/TAA	ATG/TAA
COII	ATG/TAG	ATG/TAA	ATG/TAG	ATG/TAA	ATG/TAG
ATP8	ATG/TAA	ATG/TAA	ATG/TAA	ATG/TAA	ATG/TAA
ATP6	ATG/TAG	ATG/TAA	ATG/TAA	ATG/TAA	ATG/TAA
ND5	ATT/T(AA)	ATT/TAA	ATT/T(AA)	ATT/TAA	ATT/TAA
ND4	ATG/TAA	ATG/TAA	ATG/TAA	ATT/TAA	ATA/TAA
ND4L	ATG/TAA	ATG/TAA	ATG/TAA	ATG/TAA	ATG/TAA
Cytb	ATG/TAA	ATG/TAA	ATG/TAA	ATG/TAA	ATG/TAA
ND6	ATT/TAA	ATT/TAA	ATT/TAA	ATT/TAA	ATT/TAA
ND1	ATG/TAG	ATG/TAA	ATG/TAG	ATG/TAA	ATG/TAA
COIII	ATG/TAA	ATG/TAA	ATG/TAA	ATG/TAA	ATG/TAA
ND3	ATG/TAG	ATG/TAA	ATG/TAG	ATG/TAA	ATG/TAG
ND2	ATG/T(AA)	ATG/T(AA)	ATG/T(AA)	ATG/T(AA)	ATG/T(AA)

**FIGURE 2:**
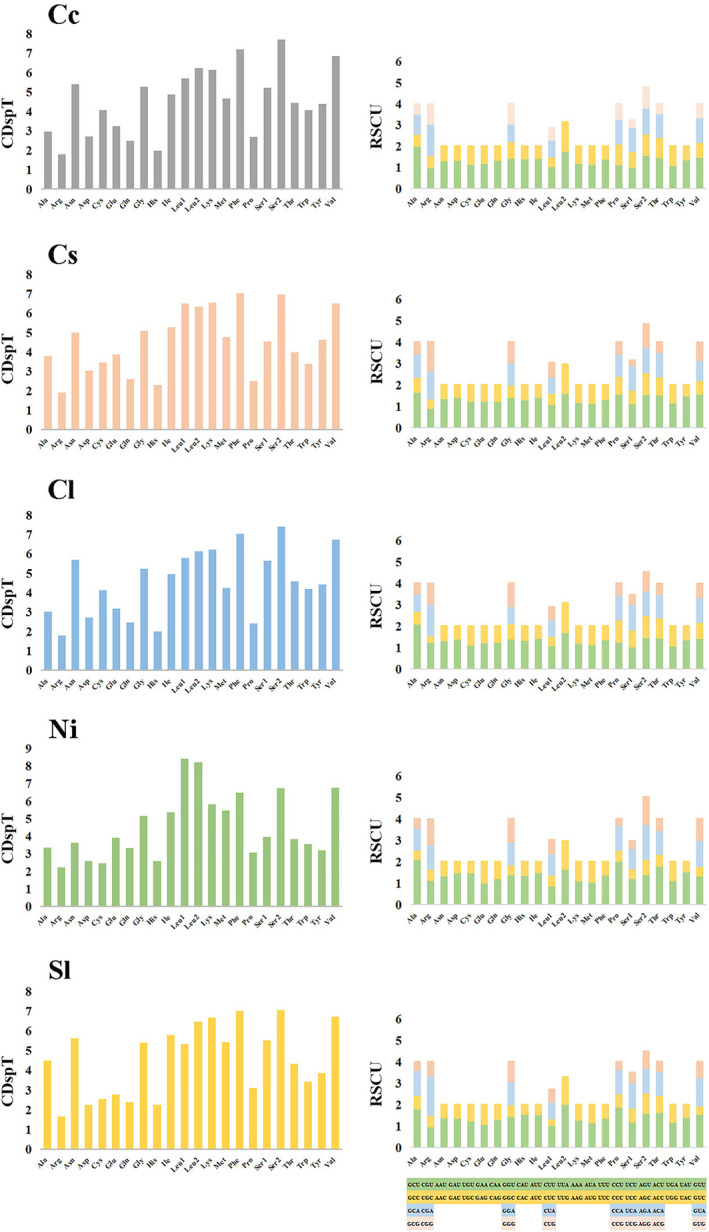
The frequency of mitochondrial PCG amino acids and relative synonymous codon usage (RSCU) of five newly sequenced neritid mitogenomes

The amino acid composition used in PCGs was relatively similar in all five species (Figure 2). The use of Leu, Lys, Ser, Phe, and Val were relatively frequent, and His and Arg were the least common amino acids. Comparing the relative synonymous codon usage (RSCU) of five species, the result showed that the average frequency of GCU (Ala), CCU (Pro), UUA (Leu2), and ACU (Thr) codons were higher than others. The amino acid content and codon usage of the 13 PCGs in these five species are similar.

### 3.3 Transfer RNAs, ribosomal RNAs, and CR

Like other complete neritids mitogenomes, there are 22 tRNA genes in these five species, including two larger regions: MYCWQGE (tRNA‐Met, Tyr, Cys, Trp, Gln, Gly, Glu) and KARNI (tRNA‐Lys, Ala, Arg, Asn, Ile) between 12S rRNA and ND3, and separated by COIII gene. The other ten tRNAs are scattered between PCGs and rRNAs (Figure 1, Table 6). The average total length of the tRNAs is 1467 bp, ranging from 56 to 72 bp (Tables 4 and 7). All of the tRNAs show significant AT base bias, with an AT content of 63.23%. The AT‐skew and GC‐skew are −0.0187 and 0.1725, respectively, showing a slight bias toward the use of T and a large bias toward C (Table 4).

**TABLE 6:** Intergenic nucleotides of five neritid species

Intergenic	Cc	Cl	Cs	Ni	Sl	Summary
COI	11	11	11	11	11	11
COII	−5	2	1	1	1	−5 to 2
tRNA^Asp^	0	0	0	0	0	0
ATP8	6	6	6	6	6	6
ATP6	31	22	28	22	22	22–31
tRNA^Phe^	0	0	0	0	0	0
ND5	0	0	0	0	0	0
tRNA^His^	0	0	0	0	0	0
ND4	2	2	2	2	2	2
ND4L	4	4	4	4	4	4
tRNA^Thr^	8	8	9	8	3	3–9
tRNA^Ser^(UCN)	5	5	5	5	5	5
Cytb	10	10	11	10	19	10–19
ND6	7	7	7	13	13	7/13
tRNA^Pro^	1	1	1	1	1	1
ND1	0	0	0	0	0	0
tRNA^Leu^(UUR)	0	0	0	14	4	0
tRNA^Leu^(CUN)	−25	−25	−25	−25	−22	−22/−25
16S rRNA	−4	−4	−8	−10	−10	−4‐(−10)
tRNA^Val^	−1	−1	−1	−1	−1	−1
12S rRNA	−1	−1	−1	−1	−1	−1
tRNA^Met^	4	4	4	5	5	4–5
tRNA^Tyr^	4	4	4	5	6	4–6
tRNA^Cys^	0	0	0	0	0	0
tRNA^Trp^	0	0	0	0	0	0
tRNA^Gln^	0	0	0	0	0	0
tRNA^Gly^	2	2	2	2	13	2/13
tRNA^Glu^	891	800	816	527	578	527–891
COIII	27	26	27	31	32	27–32
tRNA^Lys^	15	15	19	14	19	14–19
tRNA^Ala^	12	12	12	12	12	12
tRNA^Arg^	6	6	6	2	5	2–6
tRNA^Asn^	10	11	10	10	13	10–13
tRNA^Ile^	1	1	1	0	0	0/1
ND3	3	3	3	4	4	3/4
tRNA^Ser^(AGY)	0	0	0	0	0	0
ND2	99	99	99	99	99	99

**TABLE 7:** Length of the tRNAs and rRNAs of five neritid species

gene	Cc	Cs	Cl	Ni	Sl	Summary
tRNA^Asp^	66	67	66	66	67	66/67
tRNA^Phe^	66	66	66	68	68	66/68
tRNA^His^	66	66	66	66	66	66
tRNA^Thr^	68	68	68	68	68	68
tRNA^Ser^(UCN)	65	65	65	65	65	65
tRNA^Pro^	66	66	66	66	66	66
tRNA^Leu^(UUR)	68	68	68	68	68	68
tRNA^Leu^(CUN)	70	70	70	56	63	56~70
tRNA^Val^	67	67	67	68	68	67/68
tRNA^Met^	68	67	68	67	67	67/68
tRNA^Tyr^	68	68	68	68	69	68/69
tRNA^Cys^	64	64	64	65	64	64/65
tRNA^Trp^	66	66	66	67	66	66/67
tRNA^Gln^	69	69	69	69	69	69
tRNA^Gly^	67	67	68	65	65	65~68
tRNA^Glu^	66	66	66	66	66	66
tRNA^Lys^	67	67	67	67	67	67
tRNA^Ala^	68	68	68	68	68	68
tRNA^Arg^	69	69	69	69	69	69
tRNA^Asn^	72	72	72	72	72	72
tRNA^Ile^	69	69	69	69	72	69/72
tRNA^Ser^(AGY)	68	68	68	68	69	68/69
16S rRNA	1328	1333	1328	1337	1336	1328~1337
12S rRNA	865	864	865	867	868	864~868

The average length of the rRNAs is 2198 bp, with the shortest lengths of 16sRNA and 12sRNA being 1328 and 864 bp, respectively (Tables 4 and 7). These also show an AT base bias, with an AT content of 67.16%. Both the AT‐skew (0.0841) and GC‐skew (0.0405) are positive, indicating a bias toward A and G.

In the complete mitogenome of the Neritidae, the control region (CR) is the largest noncoding region, and the mitochondrial CR of all neritid species in this study was located between tRNA‐Glu and COIII, with a length of 527–891 bp (Table 6). This area usually presents a high AT bias, being an A + T rich area. This is an essential element involved in mitogenome replication and transcription initiation (Fernández‐Silva et al., 2003).

### 3.4 Ka/Ks

**FIGURE 3:**
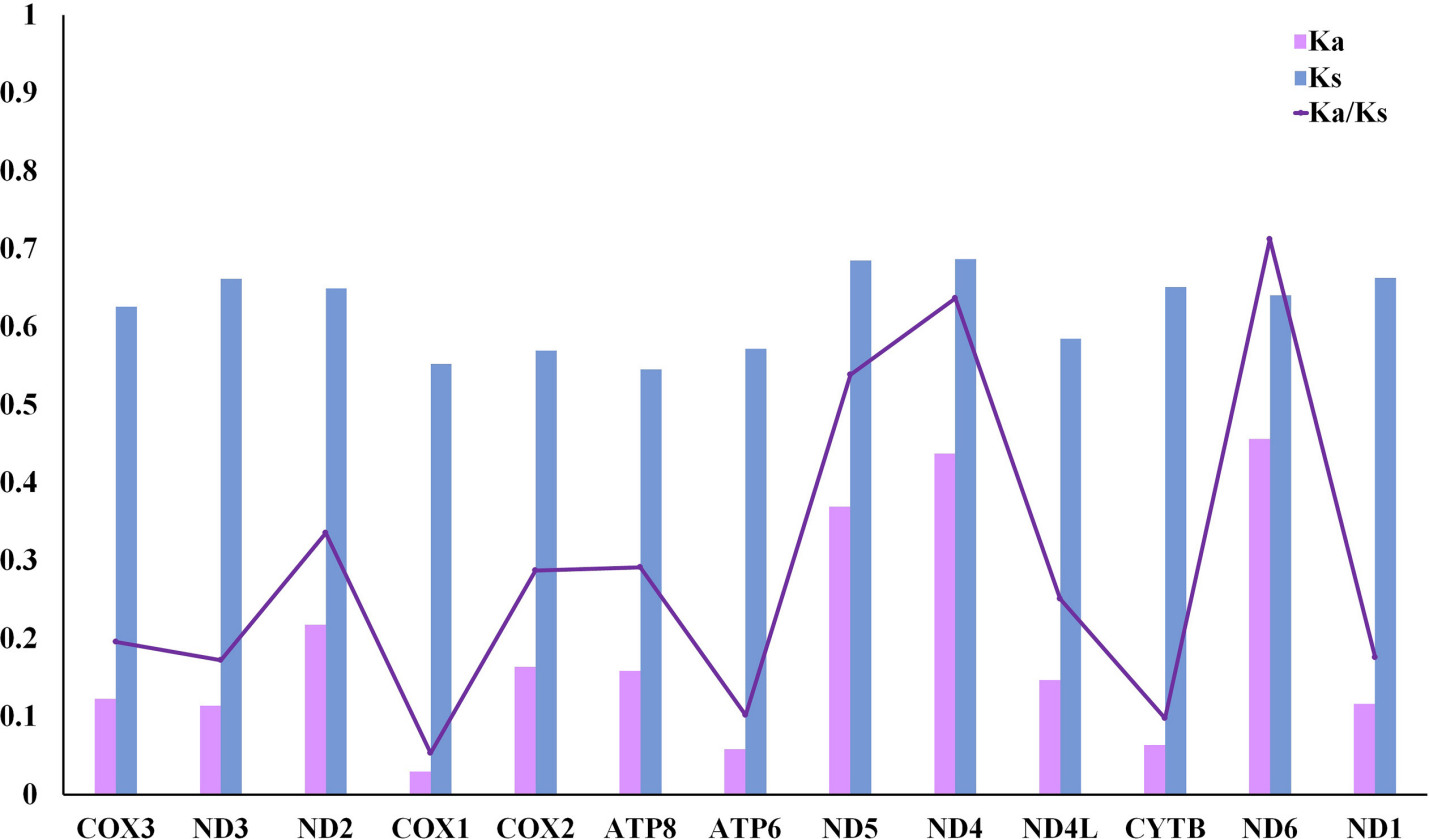
The average nonsynonymous to synonymous substitution ratio (Ka/Ks) of all 13 PCGs of 19 Neritidae species

Ka/Ks has been used as an effective way to understand the dynamic evolution of protein‐coding genes. Therefore, the Ka/Ks ratios of the 13 PCGs were calculated using the 19 sequenced Neritidae species in order to study the relationship between evolution and selection pressure (Figure 3). The results showed that the Ka/Ks ratios of the PCGs range from 0.053 for COI to 0.712 for ND6. COI has the lowest Ka/Ks value, suggesting that COI is under the lowest selective pressure to conserve the protein sequence. It is therefore widely used as a potential molecular marker in species identification and phylogenetic studies (Astrin et al., 2016).

In general, a gene is considered to be positively selected only when the Ka/Ks ratio is greater than 1. The majority of the 13 PCGs genes of the species involved in this study had relatively lower Ka/Ks ratios, ratio is less than 1. Therefore, we suggest that these PCGs may be under the influence of purification selection.

### 3.5 Phylogenetic relationships

**FIGURE 4:**
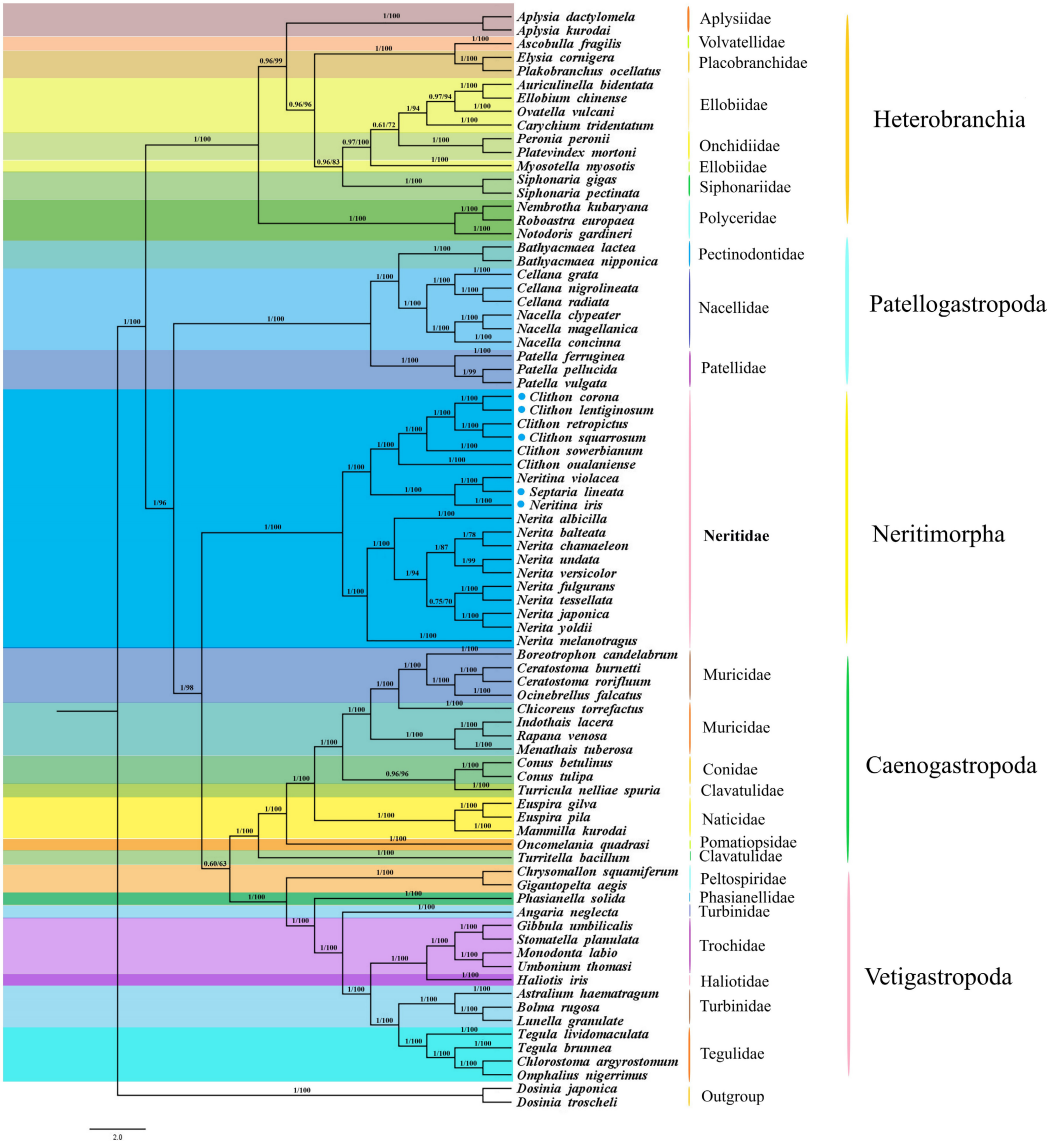
The phylogenetic tree based on 13 PCGs were inferred using Bayesian inference (BI) and maximum likelihood (ML) methods. The number at each branch is the bootstrap probability and the five newly sequenced species are marked with blue dots

The 13 PCGs of the mitogenome of 79 species from five subclasses of Gastropoda (Vetigastropoda, Caenogramopoda, Neritimorpha, Patellogramopoda, and Heterobranchia) and other two species as outgroups were used to construct phylogenetic trees (Figure 4, Table 2). The result showed that the ML tree and BI tree have a consistent topological structure, therefore, only the topology of BI tree was displayed, with strong bootstrapping for the ML tree and posterior probability values.

Our phylogenetic analysis showed that Neritimorpha is closely related to Caenogastopoda and Patellogastopoda, five subclasses within the Gastropoda show the following relationship: (((Vetigastropoda + Caenogastopoda) + Neritimorpha) + Patellogastopoda) + Heterobranchia, which was consistent with Feng et al. (2020, 2021). Kocot et al. (2011) analyze the phylogenetic relationships of Gastropoda species showing that Caenogastropoda and Heterobranchia were sister groups, and Neritimorpha is closely related to them, Patellogastropoda is on the outermost side of the phylogenetic tree. Osca et al. (2014) constructed a phylogenetic tree, finding a different result, Neritimorpha is closely related to Caenogastopoda, and then closely with Vetigastropoda. Subsequently, Uribe, Colgan, et al. (2016) added a subclass, Neomphalina, based on the research of Osca. This subclass is between Heterobranchia and Vetigastropoda in terms of evolutionary time. Zapata et al. (2014) assessed the various hypotheses that have been put forward about the inner branches of gastropod evolutionary trees in recent decades, concluding that Neritimorpha appeared on the outermost branch only once.

The phylogenetic tree of the Neritidae showed that the genus *Neritina* and *Septaria* clustered together, as a sister group with *Clithon*, the genus *Nerita* is independently distributed in Neritimorpha. According to their living habits, *Nerita* species were the only organisms widely distributed in the marine environment. Species from the genus *Neritina*, *Septaria*, and *Clithon* were common in fresh and brackish water, so they had relatively closed evolutionary relationships. Phylogenetic relationships analysis showed that all of Neritidae species were grouped together, all the posterior probability values were 1, and the bootstraps values were greater than 78. Using COI and 16s rRNA to conduct a phylogenetic tree, the results of Bunje and Lindberg (2007) show the genus *Neritina* and *Septaria* as sister groups, *Nerita* is a separate branch in the Neritidae. Chee and Mohd (2014) constructed a NJ tree using DNA barcoding of 12 species in the Neritidae, finding that *Neritina* and *Clithon* had a closed phylogenetic relationship, as sister groups with *Nerita*, this result was also consistent with recent research. Such branching results correspond to their living environment, species in Neritidae were distinguished by the difference in the salt content of the living environment.

### 3.6 Divergence times

**FIGURE 5:**
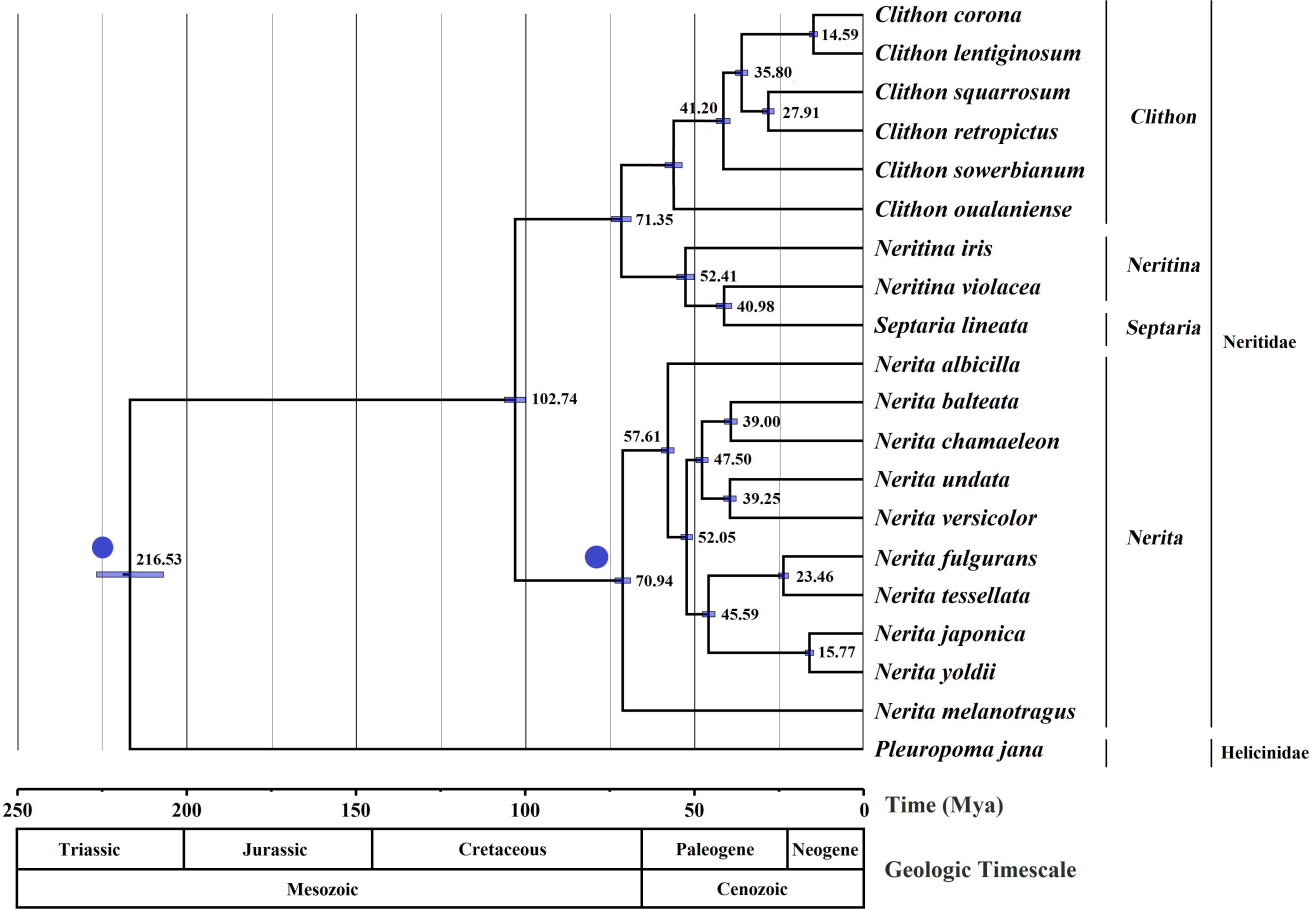
Divergence time estimations based on 13 PCGs of 19 Neritimorpha species

Our results showed that Neritimorpha originated from about 216.53 Mya (95% highest posterior density (HPD) = 206.56–226.37 Mya) (Figure 5), which is close to previous studies (Feng et al., 2020, 2021). The first divergence of the Neritimorpha was in the Triassic period, the first period of Mesozoic, which was the transition period involving the disappearance of Paleozoic biota and the formation of post‐modern biota. During this period, the marine invertebrate fauna underwent great changes (Uribe, Kano, et al., 2016). In the Neritidae, the differentiation of the four genera occurred about 102.74 Mya, the results obtained from this analysis were slightly older than the age of the origin of the Spadonidae estimated in previous reports (Feng et al., 2020, 2021). This may be due to differences between results from the fossil record and different evolutionary classification methods, which are limited by their different areas of experience and expertise. Further revision of the fossil record of the genus is needed to address the attribution of the different genera.

The genus *Nerita* was differentiated in 70.94 Mya, and other three genera were differentiated in 71.35 Mya. These five species differentiated in the Paleogene and Neogene of Cenozoic (23.03–65.50 Mya), the period of the emergence and evolution of modern organisms. The most striking effect of the Early Tertiary was the Himalayan movement: this was the period when the Qinghai‐Tibet Plateau began to rise. At this time, the continental transgression of China decreased rapidly and marine sediments appeared in the marginal areas. This crustal movement might have contributed to the rapid differentiation of the neritids during this period.

## 4 CONCLUSIONS

We sequenced the complete mitogenomes of five species in Neritidae, and analyzed basic characteristics of gene sequences, found the genome size, gene order, and nucleotide composition were similar with previous findings. The Ka/Ks ratios of 13 PCGs in 19 Neritidae species showing that these genes were under purification selection. Phylogenetic analyses indicated genus *Neritina* and *Septaria* were sister groups, and clustered with *Clithon*, genus *Nerita* was a separate branch in Nreitidae. According to the estimation of divergence times, five species differentiated in the Cenozoic. This result provides a reference for the study of phylogenetic analysis and evolution research. In this study, three of five species belong to genus *Clithon*, data from genus *Neritina* and *Septaria* are limited, further studies are needed to follow up these findings and explore the evolutionary processes of neritids.

## AUTHOR CONTRIBUTION

**Jing Miao:** Data curation (equal); Writing – original draft (equal). **Jiantong Feng:** Data curation (equal); Writing – original draft (equal). **Xiaojuan Liu:** Methodology (equal); Resources (equal). **Chengrui Yan:** Methodology (equal); Resources (equal). **Yingying Ye:** Funding acquisition (lead); Supervision (lead); Writing – review & editing (lead). **Jiji Li:** Funding acquisition (lead); Supervision (lead); Writing – review & editing (lead). **Kaida Xu:** Data curation (supporting); Writing – original draft (supporting). **Baoying Guo:** Data curation (supporting); Writing – original draft (supporting). **Zhenming Lü:** Data curation (supporting); Writing – original draft (supporting).

## CONFLICT OF INTEREST

All the authors declared no potential interest.

## Data Availability

The following information was supplied regarding the availability of DNA sequences: The complete mitogenomes of *Clithon corona*, *Clithon lentiginosum*, *Clithon squarrosum*, *Neritina iris*, and *Septaria lineata* are deposited in GenBank of NCBI under accession number MZ189741, MZ152905, MZ297477, MZ189742, and MZ315041, respectively.
